# Long-Term Storage and Longevity of Orthodox Seeds: A Systematic Review

**DOI:** 10.3389/fpls.2020.01007

**Published:** 2020-07-03

**Authors:** Svein Øivind Solberg, Flemming Yndgaard, Christian Andreasen, Roland von Bothmer, Igor G. Loskutov, Åsmund Asdal

**Affiliations:** ^1^ Faculty of Applied Ecology, Agricultural Sciences and Biotechnology, Inland Norway University of Applied Sciences, Elverum, Norway; ^2^ Nordic Genetic Resource Centre, Alnarp, Sweden; ^3^ Department of Plant and Environmental Sciences, University of Copenhagen, Taastrup, Denmark; ^4^ Department of Plant Breeding, Swedish University of Agricultural Sciences, Alnarp, Sweden; ^5^ N. I. Vavilov Institute of Plant Genetic Resources (VIR), St. Petersburg, Russia

**Keywords:** conservation, genetic resources, genebank, long-term storage, seed storage, viability

## Abstract

As part of conservation of plant genetic resources, long-term storage of seeds is highly relevant for genebanks. Here we present a systematic review and a meta-analysis of studies on seed longevity focusing on half-life (P_50_) under different storage conditions. Six studies were selected for the meta-analysis; in addition, a high number of additional references were included in the discussion of the results. The results show that under ambient conditions, half-life is short, from 5 to 10 years, while under more optimal conditions, which for orthodox seeds is at low humidity and low temperature, half-life is more in the 40−60 years range, although with large interspecies variation. Under long-term genebank conditions, with seeds dried to equilibrium and thereafter kept at minus 18−20°C in waterproof bags or jars, half-life can be twice or three times as long. In general, many of the grain legume seeds, as well as corn, common oat, and common barley are long-lived, while cereal rye, onion, garden lettuce, pepper, and some of the forage grasses are more short-lived. Conditions during maturation and harvesting influence longevity, and proper maturation and gentle handling are known to be of importance. Seed longevity models have been developed to predict final germination based on initial viability, temperature, humidity, storage time, and species information. We compared predicted germination to results from the long-term experiments. The predicted values were higher or much higher than the observed values, which demonstrate that something in the seed handling in the genebanks have not been optimal. Long-term studies are now available with data at least up to 60 years of storage. Our review shows that the knowledge and methodology developed for the conservation of plant genetic resources should also work for wild species of orthodox seed nature.

## Introduction

More than a hundred years ago, Ewart (1908) provided lists of short-, medium, and long-lived plants regarding seed longevity. This information was required because farmers and seed enterprises needed to know for how long seeds could be stored before viability seriously dropped. Results were based on experience from seed storage under ambient room conditions. Since then, various seed longevity experiments have been carried out both under ambient and cold storage conditions as well as under natural soil conditions. The Beal's soil experiments at Michigan Agricultural College (Brown, 2001; Telewski and Zeevaart, 2002) documented that some weed seeds could survive a hundred years if placed in uncorked bottles with sand buried into soil. The trial is now the world's oldest seed viability experiment started more than 130 years ago. Another pioneer study is the Vienna experiments that demonstrated a 100-years seed survival if properly stored, even under ambient temperatures (Steiner and Ruckenbauer, 1995).

E.H. Roberts is the scientist who really initiated a systematic seed research. With R.H. Ellis and their teams (e.g., Roberts, 1961; Roberts and Abdalla, 1968; Roberts, 1973; Ellis and Roberts, 1977; Ellis and Roberts, 1980; Ellis et al., 1988; Ellis et al., 1989; Ellis and Hong, 2007) pioneering seed longevity research was carried out. They showed that among abiotic factors, humidity, temperature, and oxygen are of the greatest importance; however, genetic and pre-storage factors also play a role. They also showed that orthodox seeds differ from recalcitrant seeds based on their desiccation behavior (Roberts, 1973) where orthodox seeds without damage can be dried to very low moisture contents (MCs). Chemical reactions are always dependent on water content and temperature. By further lowering the temperature, seed survival and longevity can be extended (Ellis and Roberts, 1980). Most species' seeds are of this nature and this publication exclusively considers orthodox seeds. A seed viability equation was published where the expected sigmoid germination curve was transformed to a linear curve. The equation *ν* = *K_i_* – *p*/*σ* shows the relationship between viability and storage period, where *v* is the viability after *p* years in storage, *σ* is the slope of the line, and *K_i_* is the initial viability of the seeds. The equation has been extensively applied in seed longevity and vigor studies. Their empirical data were based on seed lots exposed to different sets of artificial aging conditions. In essence, their research pointed to a uniform path of viability reduction, or that the survival curve of all seeds exhibits the same slope. They detected that the interception value varied according to genotype and pre-storage conditions. They concluded that potential storage life could be calculated based on three single factors: the seed MC, the storage temperature, and a seed lot-specific interception value. Using this equation, Ellis and Roberts could calculate longevity of any species based on information on initial seed quality. This could be done under a wide range of storage conditions. Different researches suggested improved equations, for example by Hay et al. (2003) and Probert et al. (2009). However, for simplicity, this formula is still in use, despite the large confidence intervals in the models for genebank conditions.

During second half of the twentieth century, plant genetic resource conservation became increasingly important, as did research on how to prolong seed longevity (Cromarty et al., 1982). The last evaluation of FAO (2010) showed that there are more than 1,750 genebanks (also termed seed banks) holding a total of 7.4 million accessions. The genebank standard (FAO, 2014) state that regeneration should take place before viability drops below 85% of the initial value (or when the remaining seed quantity is less than what is required for three sowings of a representative population of the accession). Regular germination tests are carried out to monitor the situation but the work is both time consuming and consume seeds. Also botanic gardens see conservation of plant diversity as one of their principal tasks, in addition to *in situ* conservation in protected areas or national parks. For these institutions seed storage under genebank conditions has been discussed as it is has been unclear that seeds of wild species store as long as the cultivated species under standard genebank conditions. The procedures for genebank storage has been standardized (FAO, 2014) and this includes the procedures for drying and packing. For genebanks drying to equilibrium in a controlled environment of 5–20°C and 10–25% RH depending upon species became standard before sealing in waterproof material stored at –18°C to –20°C (Cromarty et al., 1982; FAO, 2014). Roberts and Ellis (1989) discussed the theory of seed drying and highlighted that the water content may vary widely among species under the same drying regime due to differences in seed oil content. Proper drying would improve seed longevity, and it is said that for every 1% reduction in water content, and down to this equilibrium water content, seed longevity may double (Harrington, 1972).

The current review explores different long-term seed storage studies. Our first aim was to examine seed longevity in *ex-situ* conservation systems as they are practiced at different places. We also included references from ambient storages and from studies on the influences of pre-harvest factors on seed longevity. Our second aim was to identify species with typically long-lived or short-lived seeds among the ones with orthodox seeds and to see if there is consensus about this in the literature. Our last aim was to compare seed longevity between wild and cultivated species with orthodox seeds when such seeds are stored for more than 20 years under dry and cold conditions. This to verify if standard genebank conservation methods also works for wild species. Our overall approach has been to use long-term studies and to look for patterns rather than details, and with the focus on issues relevant for genebank management.

## Material and Methods

### Review Procedure

**Figure 1:**
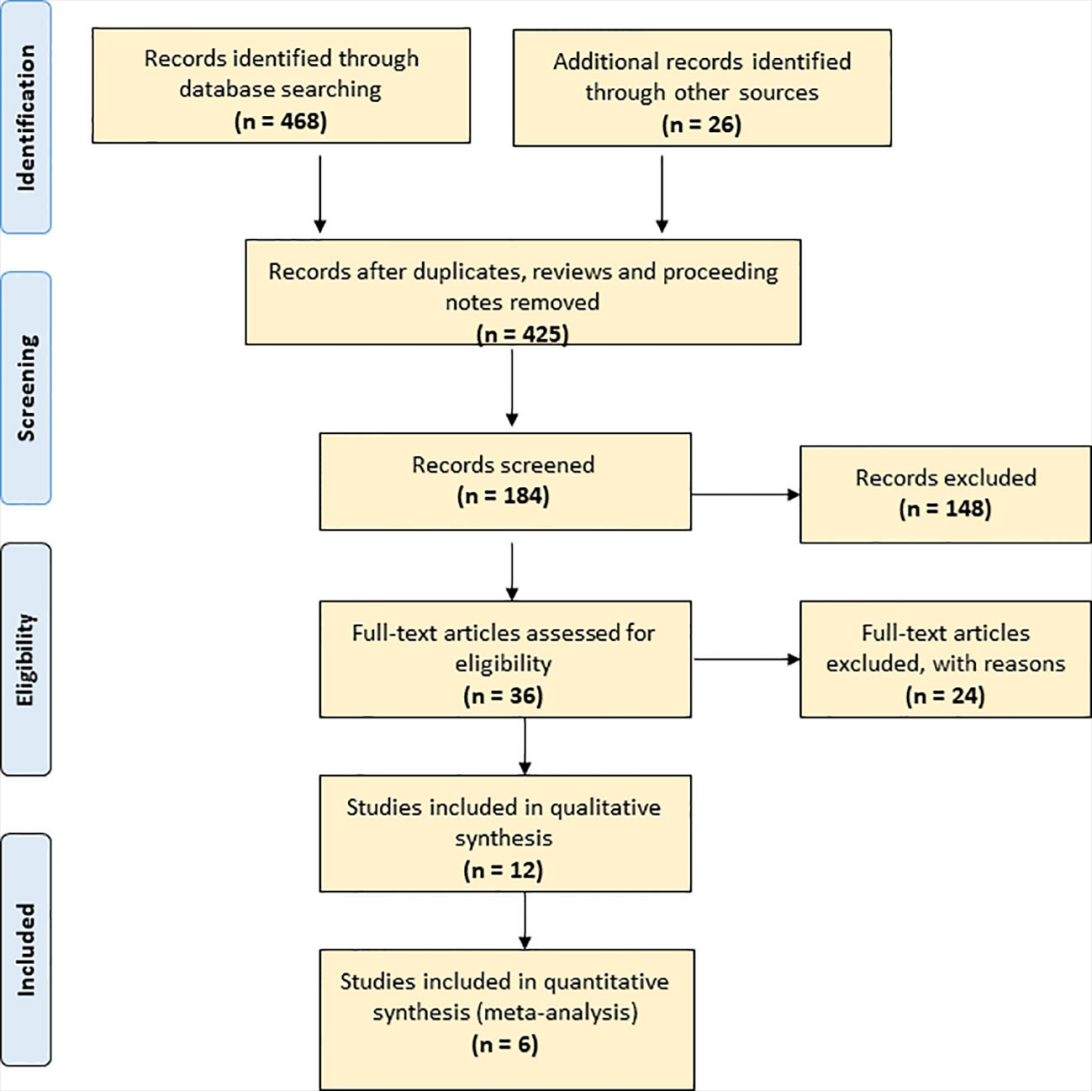
PRISMA flow diagram of the current study.

To overview publications, we applied Web of Science Core Collection. This facility covers more than 12,000 international journals from 1975 to the present. First, we used the terms “seed” and “storage” in a title search [TI = (seed AND storage)]; this yielded 468 records. We refined the search to 425 records by excluding reviews, proceedings and notes. A PRISMA flow diagram that maps out the number of records identified, included and excluded (Moher et al., 2009) is included in **Figure 1**. We further refined the search to 331 records within plant science, agronomy and horticulture and down to 184 by adding “storage” as topic [TI = (seed AND longevity) AND TS = storage]. We further refined the search to 36 by also adding “conservation” as topic [TI = (seed AND longevity) AND TS = (storage AND conservation)]. We retrieved the abstracts of the 184 articles and read the 36 articles in full text.

Among the top enhanced organizations with respect to number of records that matches our search on seed longevity on Web of Science, we found the University of Reading (UK), Royal Botanic Garden Kew (RBG Kew)(UK), The U.S. Department of Agriculture (USDA) (USA), the Indian Council of Agricultural Research (India), and International Rice Research Institute (IRRI) (Philippines). The current overview is divided into sub-sections according to storage conditions, natural soil conditions, ambient room conditions, and low temperature and humidity conditions, respectively. We used references in the examined articles to find other relevant references (“snowball method”). This was especially useful for tracing old references not provided by the Web of Science.

We also reviewed some key experiments carried out under artificial aging conditions. The experiments have played an important role in developing tools for seed longevity predictions. Regarding scientific and common names, we applied USDA, NRCS (2020). One parameter that we reviewed was P_50_ (also termed Q_50_), which is the time (in years) until the seeds in a given lot have lost 50% of their initial viability.

### Selection of Studies for a Meta-Analysis

We selected a sub-set of studies for a more thorough meta-analysis of P_50_. The selection criteria applied were: 1) datasets published, 2) storage conditions specified, 3) P_50_ details provided, and 4) a significant number of crops included. Six studies met these criteria (**Table 1**).

**Table 1:** Overview of some important seed longevity trials with seeds stored under various conditions for a given period.

Species	Storage conditions	Duration of the trial ^A^ (years)	Reference + Meta-analysis code
Natural soil condition			
21 wild species	Buried in soil, USA	120	Telewski and Zeevaart (2002)
Ambient storage			
6 vegetables	Room temp (0°C)	20	Barton (1953)
**92 crops/wild species**	**Room temp (20**°**C)**	**5−35**	Priestley et al. (1985) **, Code AM_P**
**18 crops**	**Room temp (20**°**C)**	**26**	Nagel and Börner (2010) **, Code AM_N**
Crops/wild sp.	Museum collection, room temp (20°C)	100	Leino et al. (2009)
5 oil seed crops	Ambient, +4 and −18°C	16−18	Singh et al. (2016)
Cold storage			
Forage species	Cotton bags, −15°C	20	Rincker (1983)
Cereals and weeds	Dry, room temp	100	Steiner and Ruckenbauer (1995)
**15 vegetables**	**Dry, +4/−15°C**	**20−60**	Roos and Davidson (1992) **, Code CO_R**
5 crops	Dry, –20°C or +20°C,	20	Ellis et al. (1996)
Rye	Dry, −15°C, 0°C and +10°C	17	Specht and Börner (1998)
**276 wild species**	**Dry, +4/−15°C**	**30−60**	Walters et al. (2005) **, Code CO_W**
**42 crops**	**Paper bags +5°C, < 40% RH**	**24−44**	**Stanwood (see** Walters et al., 2005 **), Code CO_S**
14 wild genera	Dry, −5°C and −10°C	32−42	Pérez-García et al. (2008)
15 wild Brassicaceae	Dry, −5°C and −10°C	40	Pérez-García et al. (2009)
157 wild species	Paper bags, +5°C	20	Chau et al. (2019)
15 crops, 41 samples	Dry, −3.5°C	30	Asdal et al. (2019)
Rice, 183 samples	Dry, +4°C and −20°C	30	Hay et al. (2013)
6 crops	Dry, 0°C and −15°C	27−34	Nagel et al. (2010)
**26 crops**	**Dry, −18°C**	**21−27**	Desheva (2016) **, Code CO_D**

Studies included in the meta-analysis are highlighted in bold.

A Duration of the viability or seed storage trial, or time the seeds have been held at the given condition before viability checks. See text for details.

For ambient storage, two studies were included:

Priestley et al. (1985, code AM_P) examined 92 species and collected storage performance data from 13 ambient storage rooms at different locations in different countries. Seeds had been stored in bags on shelves for up to 35 years. Far from all crops were present at each location and we included the data only if a crop had been present at two or more locations. Altogether 62 crops (code P1 to P62), with 169 accessions in total were included in our analysis. Mean initial germination was 98%; germination tests were performed. Observed P_50_ was interpolated based on germination data after a given storage period at each location. Species-wise P_50_ values were calculated based on averaging the location effect.

Nagel and Börner (2010, code AM_N) examined 18 agricultural/horticultural crops (code N1 to N18) stored for up to 26 years in paper bags kept under ambient conditions; +20°C and 50% RH. The study included common oat (*Avena sativa* L.), common barley (*Hordeum vulgare* L.), common wheat (*Triticum aestivum* L.), cereal rye (*Secale cereale* L.), corn (*Zea mays* L.), white lupine (*Lupinus albus* L.), common bean (*Phaseolus vulgaris* L.), garden pea (*Pisum sativum* L.), garden vetch (*Vicia sativa* L.), cabbage (*Brassica oleracea* L.), common sunflower (*Helianthus annuus* L.), common flax (*Linum usitatissimum* L.), opium poppy (*Papaver somniferum* L.), wild chives (*Allium schoenoprasum* L.), garden cucumber (*Cucumis sativus* L.), carrot (*Daucus carota* L. var. *sativus* Hoffm.), and garden lettuce (*Lactuca sativa* L.). For each species, three to five accessions were included and germination tests were conducted according to International Seed Testing Association (ISTA) protocols (ISTA, 2005) but with 50 seeds per accession. The authors calculated P_50_ based on Ellis and Roberts (1980) equation.

For cold storage, four studies were included:

Roos and Davidson (1992, code CO_R) examined viability of vegetable seeds kept for up to 60 years at the USDA plant genetic resource system (here the National Seed Storage Laboratory). The study included 69 accessions from 15 different vegetable crops (code R1 to R15). Samples were harvested from 1934 onward and were stored according to contemporary standards. The first decades (until 1977) the seeds were held in paper envelopes in metal trays at +5°C and at <40% RH; thereafter seeds were dried to equilibrium and stored at –18°C in sealed moisture-proof bags. Germination tests were conducted in 1963 and 1991, after around 20 and 50 years of storage. The crops were common bean, garden pea, spinach (*Spinacia oleracea* L.), common beet (*Beta vulgaris* L.), chard (*Beta vulgaris* L. ssp. *cicla* (L.) W.D.J. Koch), carrot, corn, garden tomato (*Solanum lycopersicum* L. var. *lycopersicum*), eggplant (*Solanum melongena* L.), okra (*Abelmoschus esculentus* (L.) Moench), garden onion (*Allium cepa* L.), pepper (*Capsicum annuum* L.), garden cucumber, cantaloupe (*Cucumis melo* L.), and watermelon (*Citrullus lanatus* (Thunb.) Matsum & Nakai). No initial germination percentages were recorded but we assumed it to be high. The authors calculated P_50_ based on Ellis and Roberts (1980), with no adjustment for initial mortality.

Walters et al. (2005, code CO_W) examined wild and cultivated species from the USDA genebank collections. The study included 276 different species (code W1 to W276) with a total number of 41,286 accessions harvested from 1963 to 1968. Seeds had been stored for 30–60 years, until 1977 under +5°C and after this at –18°C. Upon receipt at the genebank, seeds were dried to equilibrium; or a seed water content of 4–8% depending on species, and placed in metal cans that later were repacked to foil–laminate bags. Initial germination, actual germination and number of storage years was recorded and P_50_ was calculated. The authors applied the Johnson-Mehl-Avrami equation (Williams et al., 1993) to estimate P_50_. Interpolation was applied if the final germination was below 50%.

Stanwood (see Walters et al., 2005, code CO_S) initiated a study in 1977 that included 42 crops (code S1 to S42) with a total number of 207 accessions. Seeds were stored at +5°C. Seeds were dried to a seed water content of 3.7%–9.7% depending on species, and paced in envelopes, plastic vials, or cans. Germination was sampled at initiation and after 25–40 years. Species-based P_50_ values were calculated as described above by Walters et al. (2005). Interpolation was applied if the final germination was below 50%.

Desheva (2016, code CO_D) was the only study where the seeds had been kept in accordance to current long-term genebank standards. The study included 5,693 accessions from 28 species (code D1 to D28) stored for 20–24 years in the Bulgarian genebank. Seeds were dried to equilibrium at before sealed in containers at –18°C. The authors calculated the P_50_ values according to Ellis and Roberts (1980).

### Data Analysis

In our meta-analysis, we included information on initial germination, storage time, final germination, and P_50_ as given by the authors. Data were provided species-wise per study, one data-row per species but with a varying number of accessions behind. Data and species codes in the six studies are provided as supplementary material (**Table S1**). We did not weight the number of accessions in our further analysis. R software (R Core Team, 2020) was used for our statistical examination.

Initial boxplots were used to overview the distribution. We used the percentiles from the P_50_ values as provided by the authors in the studies to rank the crops per study. We ranked them into four categories, where category 1 is the highest P_50_, here defined as above the 75^th^ percentile of the given study. The 75^th^ percentile is the value below which 75% of the observations may be found. Similarly, 25% of the observations are found above the 75^th^ percentile, and here ranked in category 1. Rank category 2 is given for crops with a P_50_ between the 50^th^ and 75^th^ percentiles of that given study, while rank category 3 was given for P_50_ values between the 25^th^ and 50^th^ percentiles. Rank category 4 is the lowest and was given to P_50_ values below the 25^th^ percentile of the given study. All six studies had data on P_50_; however, not all species were present in all studies. We included species if they were present in two or more of the studies.

The four cold storage studies (CO_D, CO_S, CO_R, and CO_W) were used to analyses the correlation between parameters and to examine overall patterns and reduction in viability over storage time. The initial data were not normally distributed but even after having removed the P_50_ values above 169 years, we see some high values. The outliers and high values are from species that are still on their plateau phase and where germination has not dropped over the given storage period. Longevity models, also those used to predict the P_50_ that we examined, do not account for the often quite long plateau period during storage before viability begins to decline (Tarquis and Bradford, 1992).

Classification and regression tree (CART) are methods of nonparametric regression that evolve to overcome the difficulties with the assumptions for parametric regression. The tree technique is to extract subgroups of observations which covariates are homogeneous and between subgroups are distinct. Creation of the subgroups follow a path from the top of the tree and proceed to one of the terminal nodes (called a leaf) by following a succession of rules (called splits). The model is fitted using binary recursive partitioning whereby the data are successively split along coordinate axes of the explanatory variables so that at any node, the split that maximally distinguishes the response variable in the left and the right branches is selected. Splitting continues until nodes are pure or the data are too sparse (fewer than six cases, by default). Another advantage is that the predictor variables can be categorical as well as continuous and even a mixture of both. Modeling interactions is not a problem either. The ordinary descriptors are study code, species, storage years, number of accessions, initial germination, final germination, and P_50_ as provided by the authors of the given studies. Besides the ordinary, we created some additional ones. These were; the nature of the species (Wild of Cultivated where = W is Wild and C is Cultivar); yearly loss in germination (calculated as initial germination minus final germination divided by storage years); initial germination divided by observed germination; and P_50_ divided by observed germination.

We made a subset of our data where we included only the species where we had available details on parameters for predicting germination results by the viability equation provided by the Seed Information Database (SID) (Royal Botanic Gardens Kew, 2020). The subset included 12 species from CO_D, 13 from CO_S, 21 from CO_W, but none from CO_R study (**Table S2**). We compared the calculated predicted germination from SID to the observed germination. For calculating the SID predictions, we used species wise mean initial germination and storage years as provided by the authors of the three studies. For storage temperature, we simplified it to −18 C for the CO_D study and to +5°C for the CO_S and the CO_W study. Details on seed MC was missing in the published data except for overall values across species in two of the studies. Thus, we used MC as given by SID when dried to equilibrium. These values varied from 3.4% to 6.2% depending on species which is lower than the overall reported values.

## Results and Discussions

### The Meta-Analysis: Overall Patterns and the Effect of Storage Type

**Figure 2:**
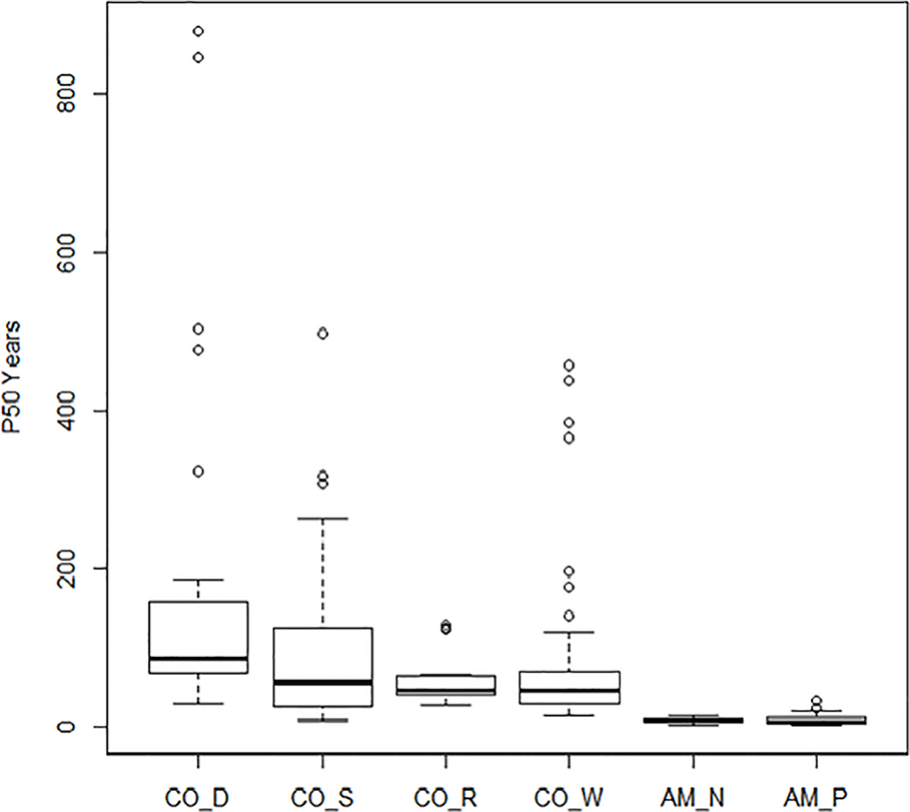
Boxplots of the estimated P_50_ (the time in years until the seeds in a given lot have lost 50% of their initial viability). Results of the four studies under cold storage: Desheva (2016): CO_D; Stanwood (see Walters et al., 2005): CO_S, Walters et al. (2005): CO_W; Roos and Davidson (1992): CO_R; and the two studies under ambient storage: Priestley et al. (1985): AM_P and Nagel and Börner (2010): AM_N.

**Figure 3:**
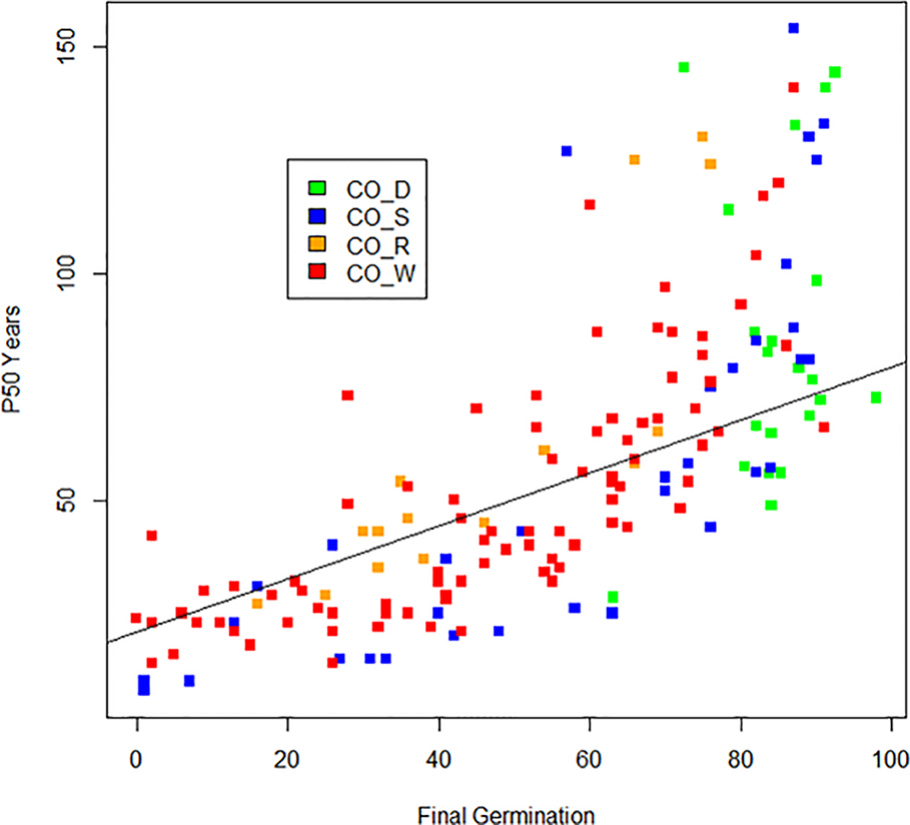
The relationship between estimated P_50_ (in number of years) and observed final germination (in %) under after long-term cold storage in the four studies (CO_D, CO_S, CO_R, and CO_W) and not adjusted for storage time. Outliers with P_50_ above 169 years were removed.

Boxplots of half-life (P_50_) are given in **Figure 2** and clearly show the influence of storage conditions on the half-life of seeds. Under ambient conditions, Priestley et al. (1985, code AM_P) showed a median P_50_ at 7 years, with 5 and 11 years as 25^th^ and 75^th^ percentiles. Nagel and Börner (2010, code AM_N) showed a similar picture. Most crops dropped in viability after 5–10 years in ambient storage. After 20 years, viability declined to nearly 0% for all crops, except for some of the cereals and grain legumes. Under cold storage conditions, P_50_ was much higher than for ambient conditions. Median P_50_ values were 56, 47, and 42 years in study CO_S, study CO_R, and study CO_W, respectively. For seeds stored according to genebank standards, which was −18°C and with dried seeds that had been packed in sealed bags (Desheva (2016), code CO_D), the P_50_ median was at 83 years with 65 and 137 years as the 25^th^ and 75^th^ percentiles (study CO_D). This is more than a ten-fold increase in longevity compared to what was found in the ambient storage studies and clearly higher than the three studies, where seeds had been kept at below +5°C for part of the storage time. We see some outliers, especially in the CO_D study with two very high values; common oat with a P_50_ of 880 years and common wheat with a P_50_ of 847 years; also, *common barley* and cultivated tobacco (*Nicotiana tabacum* L.) in the same study exhibited very high P_50_ values, with 502 and 476 years respectively. Other high values were found for mung bean (*Vigna radiata* (L.) R. Wilczek) in CO_W with a P_50_ of 457 years, finger millet (*Eleusine coracana* (L.) Gaertn.) in CO_S with a P_50_ of 437 years, and cultivated radish (*Raphanus sativus* L.) in CO_S with a P_50_ of 497 years. The P_50_ values are taken from previous studies and there are some differences between the studies in how they calculated. **Figure 3** shows the relationship between estimated P_50_ as given by the authors of the four cold storage studies (CO_D, CO_S, CO_R, and CO_W). We saw that the germination can explain 55% of variation of P_50_ in a regression analysis.

**Figure 4:**
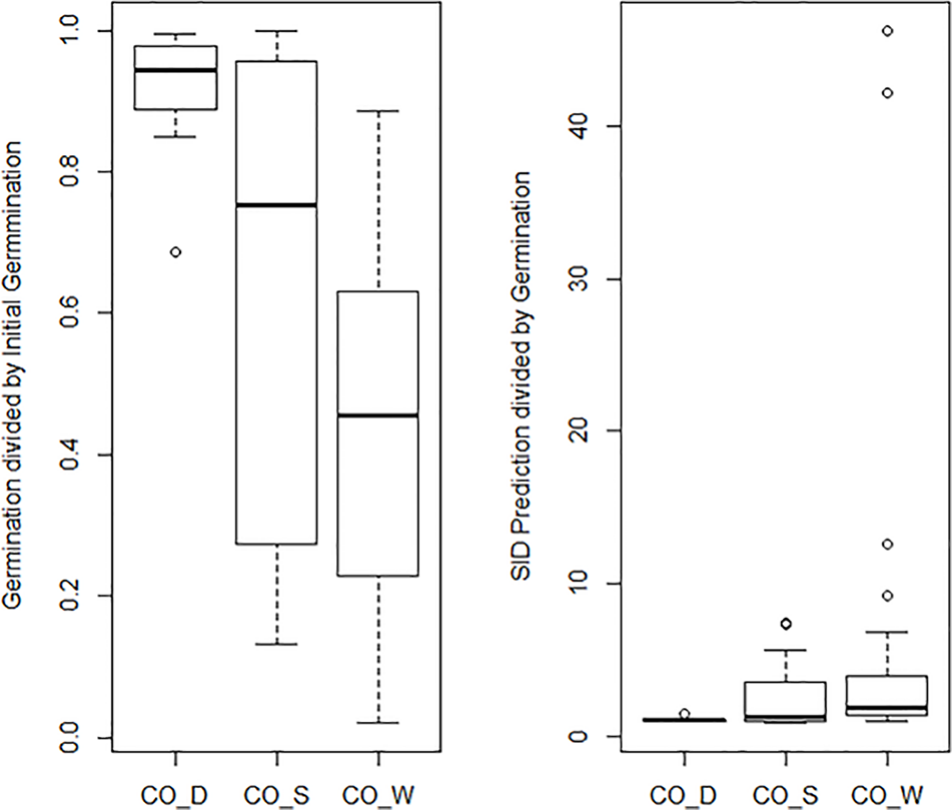
The graph to the left shows a boxplot of the final germination as proportion of initial germination for the three cold storage studies: Desheva (2016): CO_D; Stanwood (see Walters et al., 2005): CO_S, Walters et al. (2005): CO_W; Roos and Davidson (1992): CO_R. The graph to the right shows a boxplot of predicted final germination as proportion of observed final germination in the same three studies. The predicted values are extracted from the Seed Information Database (RBG Kew, 2020).

The general mean P_50_ across the four cold storage studies (CO_D, CO_S, CO_R, and CO_W) was 91.6 years. CO_D showed a P_50_ at 184 years, CO_S at 91 years, CO_R at 62 years, and CO_W at 69 years. Mean initial germination across the studies was 91.9%, mean storage time was 35.7 years, and mean final germination after the storage period was 58.4%. This gives an overall observed mean reduction in germination per year (DeltaG_Y) at 0.95% under cold storage conditions. Clear differences were, however, detected among the four studies. The observed reduction was 0.28% per year in CO_D, 1.04% in CO_S, 1.06% in CO_R, and 1.09% in CO_W. These numbers demonstrate that the storage regime has an effect, even among different cold storage systems. Different species and different number of accessions were included in the four studies. Still, we should highlight that CO_D was the only study which employed current genebank standards, with seeds dried at 10–25% RH before packed in waterproof bags and stored at –18°C. CO_S, CO_R, and CO_W had storage at +5°C for at least part of the storage time before transferred to –18°C. In addition, they had a longer or shorter history before they entered the genebank and were dried to equilibrium and sealed. For details on this we have no information. The SID longevity predictions were two to three times higher than the observed final germination values (**Figure 4** and **Table S2**). For the CO_S and the CO_W studies the SID prediction were from three to ten times higher, and in some cases more than 40 times higher than the observed final germination values. This clearly demonstrate the importance of proper seed handling also before entering genebanks.

The results from the CART analysis showed that the new calculated descriptor *Yearly loss in germination* was the most important descriptor for explaining the P_50_ (as estimated by the authors) in the four cold storage studies. Six terminal nodes were identified (**Table S3**), and all based on this same descriptor. Descriptors as *initial germination* or *final germination* were only important as inputs in the calculations but not as separate descriptors in this CART analysis of P_50_. Furthermore, the nature of the species (wild or cultivated) was not important for explaining the P_50_.

### The Meta-Analysis and Differences Among Species

Looking into each study, Roos and Davidson (1992, code CO_R) identified four plant groups: species with a P_50_ > 100 years, which included okra, garden pea, and garden tomato; species in the 50–70 years range, which included corn, eggplant, cantaloupe, and chard; species in the 30–50 years range, which included common bean, common beet, carrot, garden cucumber, spinach, and watermelon. The most short-lived species included garden onion and pepper with a P_50_ below 30 years. Walters et al. (2005, code CO_W) stressed that seeds from certain plant families were short-lived, e.g. Apiaceae and Brassicaceae, while others were long-lived, e.g. Malvaceae and Chenopodiaceae. The Brassicaceae showed an average P_50_ of 54 years, but ranged from 19 years in bladder pod (*Lesquerella palmeri* S. Watson) to 164 years in bigflower bladderpod (*Lesquerella grandiflora* (Hook.) S. Watson). The authors further stressed that correlation was detected between longevity and dry matter reserves or soluble carbohydrates of the seeds. Desheva (2016, code CO_D) showed that eleven crops had a very low decline in viability (under 5% decline compared to initial viability). These were common oat, common barley, corn, common wheat, durum wheat, smooth brome (*Bromus inermis* Leyss.), fava bean (*Vicia faba* L.), chickpea (*Cicer arietinum* L.), common sunflower, garden cucumber, and pepper. Another group, with sorghum, triticale (×*Triticosecale* Wittm. ex A. Camus), orchard grass (*Dactylis glomerata* L.), tall fescue (*Festuca arundinacea* Schreb.), common vetch, grass pea (*Lathyrus sativa* L.), lentil (*Lens culinaris* Medicus), common bean, oil-seed rape (*Brassica napus* L.), cultivated tobacco, common flax, cabbage, and garden tomato, all showing a 5–10% decline in seed viability. Larger changes, with a decline exceeding 10%, were detected for peanuts (*Arachis hypogaea* L.), garden lettuce, soybean (*Glycine max* (L.) Merr.), and cereal rye. Nagel and Börner (2010, code AM_N) showed that corn and common barley were the most long-lived with a half-life (P_50_) of around 10 years under ambient storage conditions. Among the legumes, pea was the most long-lived, with a P_50_ of 14 years. White lupine, common vetch, and common bean were also long-lived. Among the oil crops, linseed (P_50_ = 10 years) and poppy (P_50_ = 8 years) stored longer than common sunflower and cabbage. Finally, the longest-living miscellaneous crop seed was the garden cucumber (P_50_ = 14 years), followed by carrot (P_50_ = 6 years) and garden lettuce (P_50_ = 4.6 years). The lowest P_50_ value (P_50_ = only 1.9 years) was associated with chive seeds.

Regarding the two studies considering ambient storage, Priestley et al. (1985, code AM_P) showed a large variation among species. For example, many of the grain legumes exhibited the highest longevity. On the other hand, soybean—a grain legume—, as well as several grass and vegetable species, exhibited short longevity.

We liked to examine if there was agreement on which species that were long-lives or short-lived. A list was extracted from the meta-analysis and included ranking data from all six studies (see *Material and Methods*). Only species included in two or more of the six studies were included. A list of species ranging seed longevity from top to bottom is provided in **Table 2**. In the most long-lived category we found that there was consensus that pea, chickpea, mungbean, *Vicia* species, radish, and okra (*Abelmoschus esculentus*) belonged here. Also corn, sorghum, oats, barley lentils, white lupine, white clover, eggplant, and melon (*Cucumis melo*) were placed among the most long-lived species. At the bottom-end we found many of the grasses, like tall fescue, red fescue (*Festuca rubra*), timothy, and smooth brome, but also peanuts, rye, onion, pepper, lettuce, and carrot. We should highlight that our ranking across studies might have problems, especially if one or authors study only investigated short-lived or long-lived species. Furthermore, a weakness in our approach is that species may behave differently under different conditions. Therefore our rankings should be taken with such limitations.

**Table 2:** Species sorted by overall P_50_ rank, from the highest (score 1 = most long-lived, with a P_50_ above the 75^th^ percentile in a given study) to the lowest (score 4 = lowest P_50_, below the 25^th^ percentile), and where score 2 = between the 75^th^ and 50^th^ percentiles, score 3 = between the 50^th^ and 25^th^ percentiles.

Species	Rank	Respective study (code)^A^
Species with high P_50_ ranks		
*Pisum sativum*	1, 1, 1, 1	CO_R, CO_W, AM_P, AM_N
*Raphanus sativus*	1, 1, 1	CO_W, CO_S, AM_P
*Abelmoschus esculentus*	1, 1	CO_R, CO_W
*Cicer arietinum*	1, 1	CO_D, AM_P
*Vicia sp*	1, 1	CO_S, AM_P
*Vigna radiata*	1, 1	CO_W, AM_P
*Zea mays*	1, 1, 1, 1, 2, 2	CO_D, CO_R, CO_S, AM_N, CO_W, AM_P
*Avena sativa*	1, 1, 1, 3	CO_D, CO_W, AM_P, AM_N
*Lens culinaris*	1, 2	CO_W, AM_P
*Melilotus alba*	1, 2	AM_P, CO_W
*Sorghum bicolor*	1, 2	CO_S, CO_W
*Hordeum vulgare*	2, 2, 2, 1, 1	CO_S, AM_P, AM_N, CO_D, CO_W
*Cucumis melo*	2, 2, 1	CO_R, CO_W, CO_S
*Trifolium repens*	2, 2, 1	CO_S, AM_P, CO_W
*Solanum melongena*	2, 2	CO_R, CO_W
Species with high or variable P_50_ ranks		
*Solanum lycopersicum*	1, 1, 1, 4	CO_R, CO_W, CO_S, CO_D
*Spinacia oleracea*	1, 1, 2, 4	CO_W, AM_P, CO_S, CO_R
*Phaseolus vulgaris*	1, 1, 2, 2, 3, 4	CO_S, AM_P, AM_N, CO_R, CO_W, CO_D
*Cucumis sativa*	1, 1, 1, 3, 3, 4	CO_S, CO_W, AM_N, (CO_D, CO_R, AM_P)
*Citrullus lanatus*	1, 2, 3	CO_S, CO_W, CO_R
*Medicago sativa*	1, 2, 3	CO_W, AM_P, CO_S
*Poa pratensis*	1, 2, 3	CO_S, CO_W, AM_P
*Lathyrus odoratus*	1, 3	AM_P, CO_W
*Ricinus communis*	1, 4	CO_W, AM_P
*Zinnia violacea*	1, 4	CO_W, CO_S
*Nicotiana tabacum*	2, 2, 1, 3	CO_S, AM_P, CO_D, CO_W
*Triticum aestivum*	2, 2, 1, 3	CO_W, AM_P, CO_D, CO_S
*Trifolium pratense*	3, 3, 1	CO_W, AM_P, CO_S
*Beta vulgaris*	3, 3, 2, 1	CO_R, CO_W, CO_S, AM_P
Species with medium P_50_ ranks		
*Linum usitatisimum*	2, 2, 2, 3	CO_W, AM_P, AM_N, CO_D
*Dactylis glomerata*	2, 2, 3	CO_W, CO_D, CO_W
*Papaver somniferum*	2, 2, 3	CO_W, AM_P, CO_S
*Eragrostis curvula*	2, 3	CO_S, CO_W
*Fagopyrum esculentum*	2, 3	AM_P, CO_W
*Lolium multiflorum*	2, 3	AM_P, CO_W
*Lolium perenne*	2, 3	AM_P, CO_W
*Brassica oleracea*	2, 2, 3, 3, 4	CO_D, AM_P, CO_S, AM_N, CO_W
*Solanum tuberosum*	2, 4	AM_P, CO_W
*Lotus corniculatus*	3, 3, 2	CO_S, AM_P, CO_W
Species with low P_50_ ranks		
*Helianthus annuus*	3, 3, 3, 2, 4	CO_D, CO_S, AM_P, CO_W, AM_N
*Daucus carota*	3, 3, 3, 2, 4	CO_W, AM_P, AM_N, CO_S, CO_R
*Pennisetum glaucum*	3, 3	CO_W, CO_S
*Phleum pratense*	3, 3	CO_W, AM_P
*Allium ampeloprasum*	3, 3, 4	CO_W, AM_P, AM_N
*Onobrychis viciifolia*	3, 3, 4	CO_W, AM_P, CO_S
*Brassica napus*	4, 4, 1	CO_D, CO_W, AM_P
*Bromus inermis*	4, 4, 1	CO_W, AM_P, CO_D
*Apium graveolens*	4, 4, 2	CO_W, AM_P, CO_S
*Lactuca sativa*	4, 4, 4, 1, 3	CO_W, CO_S, AM_N, CO_D, AM_P
*Petroselinum crispum*	4, 4, 2	CO_W, AM_P, CO_S
*Capsicum annuum*	4, 4, 2, 3	CO_W, CO_R, CO_D, CO_S
*Festuca rubra*	4, 4, 3	CO_W, AM_P, CO_S
*Allium cepa*	4, 4, 4, 3	CO_R, CO_W, CO_S, AM_P
*Secale cereale*	4, 4, 4, 3	CO_D, AM_P, AM_N, CO_W
*Arachis hypogaea*	4, 4	CO_D, CO_W
*Crambe abyssinica*	4, 4	CO_W, CO_S
*Festuca arundinacea*	4, 4	CO_S, AM_P

Only species included in two or more studies are listed, the given code is provided in the same order as the rank score.

A AM_P = Priestley et al., 1985; AM_N = Nagel and Börner, 2010; CO_S = Stanwood (see Walters et al., 2005); CO_R = Roos and Davidson, 1992; CO_W = Walters et al., 2005; CO_D = Desheva, 2016.

### Other Studies Examining the Effect of Storage Conditions

Leino et al. (2009) examined samples of cereals, grain legumes, and other agricultural crops from a seed collection maintained at the Swedish Museum of Cultural History with seeds dating from 1862 to 1918, which means they were around 100 years old. None of the samples germinated. Leino and Edqvist (2010), however, succeeded in germinating 151 year-old *Acacia* seeds collected in Egypt in 1856 and subsequently stored at room temperature in a Swedish museum. They reviewed studies from other museum collections and found evidence of seeds of wood species (in particular) surviving ambient conditions for more than 100 years. This does not mean that such longevities are common, but that there are examples of very long-lived seeds. Steiner and Ruckenbauer (1995) published another example of museum samples that could survive for a long time. They examined the viability of dried seeds (dried to approximately 3% internal MC) of cereals and weed species stored hermetically sealed and kept at +10°C to +15°C for 110 years (the Vienna samples of 1877). The initial germination percentages were unknown, but assumed to be high. After 110 years of storage, common barley and common oat still had 90% and 81% germination respectively. Seeds of some weed species were also germinating but at low germination percentages. These included the weeds cockle (*Agrostemma githago* L.), bearded ryegrass (*Lolium temulentum* L.), field mustard (*Sinapis arvensis* L.), and bladder-soapwort (*Vaccaria hispanica* (L.) Sm.), as well as the cultivated plant white mustard (*Sinapis alba* L.). This study clearly demonstrated that dry, long-term storage under ambient temperature conditions is possible. However, keeping seeds on shelves in more or less open bags is not the same as the Vienna samples. Barton (1953) examined six vegetables stored for 20 years at ambient conditions (20°C). For comparison, samples were stored at minus 4°C in bags (internal humidity not reported). Crops included were carrot, eggplant, garden lettuce, garden onion, pepper, and garden tomato. After 20 years under ambient conditions, seeds of carrot, eggplant, and garden tomato were viable but had low germination percentages. Garden lettuce, garden onion, and pepper seeds were all dead. If stored at −4°C in sealed bags, all crops were still viable; some had germination percentages of around 90%. At the end of the 20-year study period, the study terminated. Another study under ambient condition, which also included cold room storage, was published by Singh et al. (2016). The study examined chickpea, sesame (*Sesamum indicum* L.), safflower (*Carthamus tinctorius* L.), castor bean (*Ricinus communis* L.), and ramtilla (*Guizotia abyssinica* (L. f.) Cass.). At ambient temperature, they concluded that seeds lost the ability to germinate within one to 12 years depending on crop and MC. Furthermore, without proper drying (6−9% internal MC), the storage time was a single year for sesame, 4 years for castor bean and safflower, 9 years for chickpea, and 12 years for ramtilla, while at +4°C and dried to an equilibrium at 15% RH, germination was 94% for ramtilla, 88% for safflower and chickpea, 86% for sesame, and 36% for castor bean. At −18°C and dried to an equilibrium at 15% RH, no significant decline in germination was recorded except for sesame (Singh et al., 2016).

van Treuren et al. (2018) carried out an experiment in the Netherlands in common wheat and common barley stored at +4°C and −20°C. Initial viability was at 94−95%. After 23–33 years, the viability remained as high as 90−94% for the seeds stored at −20°C. Samples stored at +4°C showed 62% germination for common wheat and 75% for common barley. Hay et al. (2013) also examined seed survival at +4°C and at −20°C storage but they used rice as study plant. At −20°C storage, germination remained above 70% in most samples while at +2 to +4°C storage, results were much more variable. They predicted P_50_ to vary from 50 to 900 years depending on storage conditions and initial seed quality. A third experiment, with +10°C, 0°C, and −15°C storage was carried out by Specht and Börner (1998) using rye. Seeds were dried to internal MC of 3.7–5.5% and hermetically sealed in glasses with air, CO_2_, and N_2_. Initial germination percentage was 72%. After 17 years, germination had dropped significantly at the +10°C storage in contrast to the −15°C storage. Overall, N_2_ reduced the germination losses compared with air.

Before summing up this section, we should refer to an experiment carried out by Ellis et al. (1996). They examined seeds of carrot, peanut, garden lettuce, oilseed rape, and garden onion kept for 5 years at two different MC regimes, combined with +20°C or −20°C respectively. No loss in seed viability was detected during this period in any of these species at −20°C in either moisture regime. Significant loss in viability occurred at +20°C. The loss was faster in seeds that had been dried to a MC of 5.5–6.8%, than in seeds that had been dried to 2.0–3.7% MC. The same pattern was observed in all five species.

### Other Studies Showing Differences Among Species

The short-lived nature of some of the species ranked at the lower end has also been demonstrated in other studies. Barton (1953) mentioned both garden lettuce, garden onion, and pepper in this category. Additionally, Ozcoban and Demir (2002) showed that pepper is especially short-lived; Nagel and Börner (2010) showed the same for garden lettuce and garden onion. Lu et al. (2018) also documented that garden lettuce is short-lived. As part of their examination of more than 18,000 accessions from 23 crops in the National Gene Bank of China, five crops showed a decline in germination that was clearer than for the others; carrot, garden lettuce, cotton (*Gossypium* sp.), common flax, and castor bean; however, the decline was especially marked for carrot and garden lettuce.

The long-lived nature of common barley and corn, as well as the fact that cereal rye is short-lived, have also been reported by Nagel et al. (2010), who examined common barley, common wheat, cereal rye, sorghum (*Sorghum bicolor* (L.) Moench), oilseed rape, and common flax with 40–50 accessions per crop. The seeds were stored for around 30 years at −15°C in accordance with contemporary standards, with seed MCs of ± 8%. Common wheat, in particular, showed a very high variation in viability, ranging from 0% to 87% germination after 34 years of storage. Common barley and common flax showed a tendency to longevity while cereal rye and oilseed rape were more short-lived. All cereal rye accessions and most of the oilseed rape accessions had less than 50% germination after 30 years. Asdal et al. (2019) have also confirmed the short-lived nature of cereal rye. They presented the results of the first 30 years of the 100-year experiment in permafrost (−3.5°C) at Svalbard. The study included 17 crops with two to three samples per species, and seeds were dried to 5−7% seed water content before being sealed in glass containers. The results showed that mean germination across all test samples dropped by 10.3% over the first 30 years, from 87.2% at year 0 to 76.9% at year 30. At the lower end of the scale, rye had lost 51% of the initial germination percentage. Among the other cereals, common barley showed the highest viability at 89%, while common wheat retained 79% of initial germination percentage. In contrast to our meta-analysis, Asdal et al. (2019) reported good viability for garden lettuce, carrot, and garden onion.

### Wild Species Versus Cultivated Species

**Figure 5:**
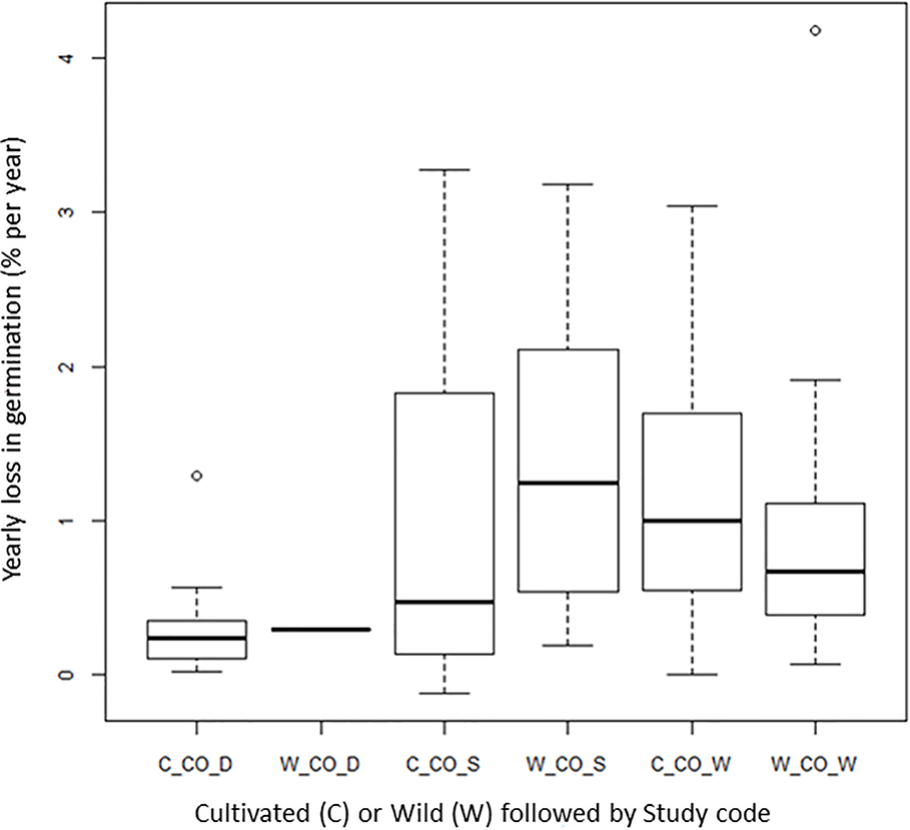
Average yearly decline in germination (DeltaG_Y) for cultivated (prefix C-) and wild (prefix W-) material in the given long-term studies under cold storage: Desheva (2016): CO_D; Stanwood (see Walters et al., 2005): CO_S, Walters et al. (2005): CO_W; Roos and Davidson (1992): CO_R.

One question we had was if wild species shows as good longevity as cultivated species under genebank storage systems, a question raised by Colville and Pritchard (2019). Three of the four long-term studies had both wild and cultivated species (CO_D, CO_S, and CO_W). Our analysis shows no clear differences in seed longevity between wild species and cultivated species (**Figure 5**). The CART analysis also showed that the wild species are found in different terminal nodes, confirming no clear differences between the two categories of plants (**Table S3**). We are aware that there are different wild species included in the different experiments and that there is not a balanced number of species and accessions that we have compared. Still, the overall pattern shows that wild species can stand genebank conservation system and be a complement to *in-situ* conservation of wild species. If we look at other studies with wild species, Pérez-García et al. (2008) examined 14 accessions from genera of threatened, endemic species from different families. The seeds, one accession from each genus, were dried, sealed, and stored for 32−42 years at −5°C to −10°C before germination was tested. In 10 of the 14 accessions, germination was 90% or higher. In two accessions, it was around 70% and two had values below 55%. A tetrazolium test showed that these accessions also had dormant seeds and that the viability was higher than 55%. The authors concluded that using drying methods for long-term storage of orthodox seeds is useful for *ex situ* conservation of a range of wild species across various plant families. Pérez-García et al. (2009) also examined the longevity of 15 wild species of Brassicaceae stored under the conditions given above for 40 years. Overall, these seeds also survived very well. The poorest result was found for spiny alyssum (*Hormatophylla spinosa* (L.) Küpfer) with a 7% decline over the 40 years of storage.

Regarding seed survival in soil, results are more contradictory. Nascimento and Meiado (2017) showed a very short survival of seeds in soil compared to seeds kept in paper bags in a cold chamber. They examined a few wild species. Germination was evaluated monthly for about 2 years. During the first 10 months, no difference was found between seeds conserved *in situ* and *ex situ* and all seeds germinated well. After 1 year, the differences became clear and at the end of the experiment, the buried seeds exhibited almost complete loss of viability while the seeds stored in the cold chamber continued highly viable. On the other hand, the Beal's experiment demonstrated that a few wild species as moth mullein (*Verbascum blattaria* L.) and low mallow (*Malva rotundifolia* L.) can survive a hundred years in soil (Brown, 2001; Telewski and Zeevaart, 2002).

### The Importance of Pre-Harvest Factors

In a study by Lu et al. (2018), more than 18,000 accessions from 23 crops in the National Gene Bank of China were tested after a decade in storage. In general, germination was good, but in some accessions—0.5% of the total number—, germination had declined to 70% or below. Intraspecific variations have been reported in other studies as well (e.g., Balouchi et al., 2017; Asdal et al., 2019) and Nagel et al. (2009) were able to identify quantitative trait loci (QTLs) for seed longevity. They examined common barley and could locate such genes to chromosomes 2H, 5H, and 7H. Therefore, seed longevity may vary among cultivars; there are also other important factors. Rao et al. (2017) provided a recent overview of such factors. Among abiotic factors, humidity and temperature are of the greatest importance but there are also genetic and pre-storage factors involved (Bewley et al., 2013; Zinsmeister et al., 2020). Proper ripening is important (Dias et al., 2006). In general, immature seeds loose viability faster than mature seeds (Austin, 1972; Justice and Bass, 1978). For example, for cereals potential seed longevity is highest if harvested about 2 weeks after end of grain filling (Ellis and Filho, 1992; Ellis et al., 1993; Rao and Jackson, 1996a), or around the time when cereals are combine-harvested (Filho and Ellis, 1991). Harvest delayed beyond optimum maturity can contribute to rapid deterioration, especially in humid environments (Rao and Jackson, 1996b). Hence, when maximum quality is reached, and how long it is maintained during seed development and maturation, varies with genotype and environment (Ellis, 2019). Climate during maturation, harvesting method, and the presence of pests and diseases further influence the result (Austin, 1972).

Regarding other physical factors related to the storage environment, Groot et al. (2015) showed that the ageing of dry seeds was accelerated by the presence of oxygen in the storage environment and recommended anoxic conditions to prolong seed longevity.

Zhou et al. (2019) showed how endogenous factors such as the structure of the seed coat but also the balance of hormones, nucleic acids, and proteins are involved in controlling seed longevity. Damage to seeds during harvesting may also be of importance. Rincker (1983) examined longevity of forage crops stored in freezers at −15° C for 20 years. Seeds were placed in cotton bags without any equilibrium drying or sealing mechanisms. The study included alfalfa (*Medicago sativa* L.), red clover (*Trifolium pratense* L.), white clover (*T. repens* L.), alsike clover (*T. hybridum* L.), birdsfoot trefoil (*Lotus corniculatus* L.), smooth brome, orchardgrass (*Dactylis glomerata* L.), timothy (*Phleum pratense* L.), and perennial ryegrass (*Lolium perenne* L.). Germination was tested initially at the end of the experiment. Mean germination across all species declined by less than 2% over these 20 years. Red clover, smooth brome, orchardgrass, and timothy retained viability better than alfalfa, where big variation among seed lots was detected. The variation could be explained by location and/or thresher-damages during alfalfa seed production. Niedzielski et al. (2009) suggest that more traits are involved in the expression of seed longevity than initial viability and other parameters typically measured in studies of seed vigor. Safina and Filipenko (2013) reported the same findings in connection with the artificial ageing of winter wheat, common bean, and peas. The ageing depended not only on initial viability and seed MC, but also on the seeds' genetic features.

Probert et al. (2009) examined 195 wild and cultivated species under artificial aging conditions to find patterns in seed longevity. Species in 71 different plant families from all around the world were included and P_50_ was determined at +45°C and 60% RH. Depending on species, P_50_ varied from less than 1 day to more than 2 years under the given sub-optimal conditions. In general, endospermic seeds were more short-lived than non-endospermic seeds and seeds from plants adapted to cool and wet conditions were more short-lived than seeds from plants adapted to hot and dry environments. Seed weight and oil content were not correlated with longevity. They concluded that the apparent short-lived nature of endospermic seeds from cool and wet environments would have implications for *ex situ* conservation strategies. Merritt et al. (2014) examined 172 Australian wild species and found many of the legume (*Fabaceae*) and myrtle (*Myrtaceae*) species to be long-lived. Mondoni et al. (2011) examined 63 alpine species from northern Italy. Compared to the species from Australia, alpine species were more short-lived.

### Seed Longevity and Genebank Management

Long-term and medium-term storage of seeds at low temperature serves as a safe and relatively inexpensive method of plant genetic resources conservation and the importance of temperature on seed longevity is well documented in several genebank studies. For example, a system of low-temperature storage at the VIR genebank in Russia began in the 1950s with a temperature of +4°C. Seeds were put into glass bottles and sealed. At present more than 260,000 seed samples of cultivated plants and their wild relatives are maintained this way (Filipenko, 2007). More recently, −20°C storage facilities have been built for long-term storage. The value of a proper drying is also well-known. Drying is usually achieved using dehumidifiers but an alternative approach could be to use silica gel (Filipenko et al., 2018).

We mentioned that one criterion in international genebank standards is to commence regeneration before viability declines to 85% of its initial value (FAO, 2014). If we look at the results from our four studies under cold storage, germination percentages decreased in the range of 0.95% per year, or a reduction from 100% to 85% within less than 20 years. This is far higher than predicted in many theoretical studies applying viability equation.

With high initial seed quality and standard genebank storage conditions, a 5% reduction in viability should theoretically take 70–80 years for common barley and common wheat, more than 1,000 years for pea, 28 years for garden onion, and 11 years for garden lettuce (Ellis and Roberts, 1977; Ellis et al., 1988; Ellis et al., 1989). Our meta-analysis shows that these estimates may be too optimistic. In practice, genebank conditions may not be as optimal as claimed, or seed quality may not be optimal. An online tool based on Ellis and Roberts' research has been developed (Royal Botanic Gardens Kew, 2020). The model indicates general expectations and is less useful for single seed lots. As our review has shown, each seed lot is unique but within boundaries of the species and the storage conditions. Still, genebank managers want to have indications on when a specific sample will fall to 85% of its initial germination (without using up the seeds for periodic germination tests). Although the models are well grounded, the confidence intervals are large when it comes to predicting storage time under dry and cold conditions. Some verifications of the viability equation have been made but these are mainly under artificial aging conditions, e.g. by Dickie et al. (1990) who examined very different species, ranging from common barley, garden lettuce, and grain legume to field elm (*Ulmus carpinifolia* Gleditsch.). Kraak and Vos (1987) examined seeds of two garden lettuce cultivars stored with MCs ranging between 3.6% and 17.9% (fresh weight basis) at constant temperatures ranging between +5°C and +75°C. They observed that more than 94% of the variation at the restricted temperature range of 5–40°C could be explained by a simplified equation assuming either a log-linear relationship between seed longevity and temperature, or a log-linear relationship between seed longevity and both MC and temperature. Nevertheless, the uncertainty indicated by linear models may be too high (Sapra et al., 2003). Although refining the existing models is interesting, it will only continue to document the problem that genebanks managers have. Here artificial aging tests can be used to separate lots on the basis of their potential longevity (Bradford et al., 1993) and be used in predicting how they will behave under other conditions using the viability equation. If small artificial aging test were carried out under standard conditions of all lots, this information would give relative rankings of these lots. Over time, under current protocols, their actual longevity under the storage conditions could be revealed. It will then be possible to relate the initial characterization of the seed lot's potential storability with its actual longevity under the genebank conditions and give a fairly good relative estimate of the length of the initial plateau period before longevity starts to fall rapidly. Such deterioration tests will be a guide for managers on how long they can wait without waiting too long with germination tests. At a minimum, it will allow calibration of the former by the latter. This adds work to the seed testing lab, but it would also greatly reduce the routine tests being done when viability is still high, using up seeds but providing no predictive information. However, not to carrying out germination tests is risky, as some accessions may drop in germination very fast and where the artificial aging test is no guarantee. But continuing to do more of these post-storage, retrospective studies without comparable pre-tests will never solve the essential problem of knowing which lots will need regeneration soon and which will last much longer.

### Concluding Remarks

Seed longevity is a complex issue where collecting evidence is both time-consuming and complex. Our first aim was to examine how fast germination reduction in long-term storage under different conditions. Our review and meta-analysis showed that germination dropped slowly if seeds were dried to equilibrium before sealed and stored at low temperature. The loss in germination was much faster if seeds were handled sub-optimal, like keeping the seeds at 40% and +5°C for some years before properly dried and conserved. We have identified that overall germination loss across species is in the range of 0.2–0.3% per year if stored under the recommended genebank conditions, however more data is needed to verify this number. A higher loss was found in our examined long-term studies than what can be predicted to be the loss using the seed viability equation as provided by Ellis and Roberts and the SID database, even if seeds were dried and stored under optimal conditions. There are of cause species variation, but this overall pattern tells us that there is more to be explored, either practically or theoretically within the field of seed longevity.

Our second aim was to identify if there were typically long-lived or short-lived species, and if there is consensus about this. We grouped the species into four categories based on our meta-analysis and found agreement for several of the species across the studies. Most long-term storage data are from germination monitoring trails in genebanks. These data are from seeds stored for up to 60 years. What we have seen is that many species can survive good storage conditions for 30−50 years without any serious drop in viability while others lose viability very quickly. Under ambient or more natural soil conditions, viability drops considerably within a few years. Several studies have shown the importance of seed quality for storage performance. Many demonstrate the importance of storage temperature and seed humidity, as well as the role of species variation and pre-harvest and proper maturation conditions. We have systematically examined long-term experiments and found variable results. Predictions based on artificial aging say that seeds can be stored for hundreds of years without seriously affecting viability, as long as initial viability is good and seeds are kept at low temperature and humidity. International genebank standards state that viability should be monitored for each seed sample every tenth year. If a sample maintain viability for a hundred years or more, such frequent sampling seems a little excessive. For example, at Centre for Genetic Resources, the Netherlands at Wageningen the recommendation is to delay the first germination tests to 25 years post-storage for materials applying standard procedures and storage conditions (van Treuren et al., 2013). It is therefore interesting to follow long-term experiments. To date, the available information is relatively limited and published data is of great value. We recommend collaboration among genebanks in sharing longevity data. Globally agreed standards for long-term storage dictate proper drying, sealing and storing at about −18°C. There is no doubt that these conditions significantly extend seed life span compared to ambient or refrigerated storage. Regarding our final aim to compare seed longevity between wild and cultivated species under genebank storage conditions, we found no clear differences, which means that this type of *ex-situ* conservation also works for wild species. This is an important result as genebanks for wild species most likely will be of higher importance in the future and work as a supplement to in-situ conservation of threatened species and crop wild relatives.

## Data Availability Statement

All datasets presented in this study are included in the article/**Supplementary Material**.

## Author Contributions

SS was the lead writer. FY analyzed the data. RB, ÅA, IL, and CA contributed with ideas and edits.

## Funding

Our study was developed with support from NKJ − the Nordic Joint Committee for Agricultural and Food Research under the Nordic Council of Ministers.

## Conflict of Interest

The authors declare that the research was conducted in the absence of any commercial or financial relationships that could be construed as a potential conflict of interest.

## Supplementary Material

Table S1 Supplementary file with data from the meta-analysis showing all species, number of accessions per species, initial germination percentage, storage years, final germination percentage, average yearly changes in germination and observed/estimated P_50_ from each of the studies included. See text for explanation.

Table S2 Supplementary file with predicted final germination data from the Seed Information Database from RBG Kew compared to observed final germination data from the given long-term studies included in our meta-analysis. See text for explanation.

Table S3 Supplementary file with data for the different terminal nodes in the tree regression analysis (last column). Species information, number of accessions per species, initial germination percentage, storage years, final germination percentage, average yearly changes in germination and observed/estimated P_50_ from each of the studies included. See text for explanation.
